# Supplementary material for: Microfibril-associated glycoprotein 4 forms octamers that mediate interactions with elastogenic proteins and cells
Source: Nat Commun. 2024 May 13;15:4015. doi: 10.1038/s41467-024-48377-z (PMC11091212; doi:10.1038/s41467-024-48377-z)
Supplement: Supplementary file 1 — Supplementary Information [file 41467_2024_48377_MOESM1_ESM.pdf]

SUPPLEMENTARY INFORMATION

Microfibril-associated glycoprotein 4 forms octamers that mediate interactions with elastogenic proteins and cells

Michael R. Wozny[1,*], Valentin Nelea[1,2,*], Iram Fatima S. Siddiqui[1], Shaynah Wanga[3], Vivian de Waard[3], Mike Strauss[1,#], Dieter P. Reinhardt[1,2,#]

[1] Faculty of Medicine and Health Sciences, McGill University, Montreal, QC, Canada

[2] Faculty of Dental Medicine and Oral Health Sciences, McGill University, Montreal, QC, Canada

[3] Amsterdam UMC location University of Amsterdam, Medical Biochemistry, Amsterdam, The Netherlands; Amsterdam Cardiovascular Sciences, Amsterdam, The Netherlands

* These authors contributed equally

# These authors jointly supervised this work

Corresponding authors: Dieter P. Reinhardt (dieter.reinhardt@mcgill.ca) and Mike Strauss (mike.strauss@mcgill.ca)

Supplementary Figure 1

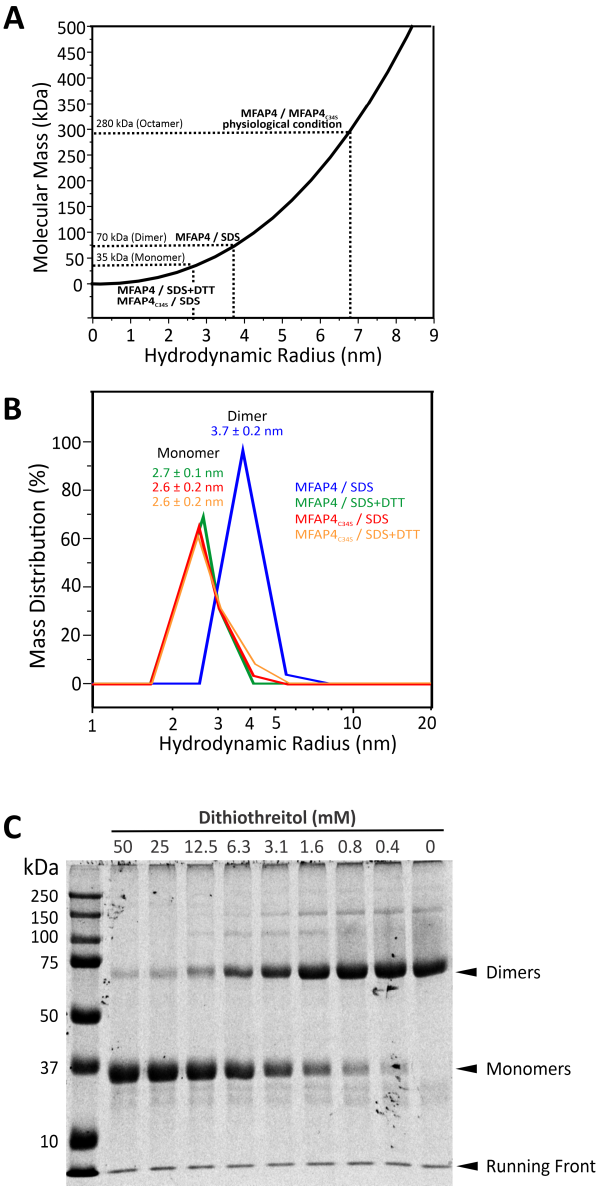

Supplementary Figure 1. MFAP4 multimers dissociate into dimers under denaturing conditions and monomers under reducing conditions. (**A**) The curve shows theoretical hydrodynamic radii plotted against molecular masses of globular proteins. Measured hydrodynamic radii for MFAP4 and MFAP4$_{C34S}$ are shown (dashed lines) in the presence or absence of 1% SDS or 50 mM DTT as indicated. (**B**) DLS-determined hydrodynamic radii of MFAP4 (blue and green) and MFAP4$_{C34S}$ (red and orange) under denaturing conditions in the presence of 1% SDS in TBS buffer either with or without reducing DTT (50 mM) as indicated. Shown is one representative experiment measured in technical triplicates of a total of n=3 independent experiments with similar results. Means and standard errors of the mean of hydrodynamic radii derived from all three experiments are indicated. (**C**) MFAP4 was treated with decreasing concentrations of DTT (50-0 mM) in physiological buffer (TBS/Ca^{2+}), alkylated, and analysed on SDS-PAGE as described in the Methods. The experiment was performed two times with identical results. Arrowheads indicate the positions of MFAP4 monomers and dimers, as well as the running front. Source data are provided as a Source Data file.

Supplementary Figure 2

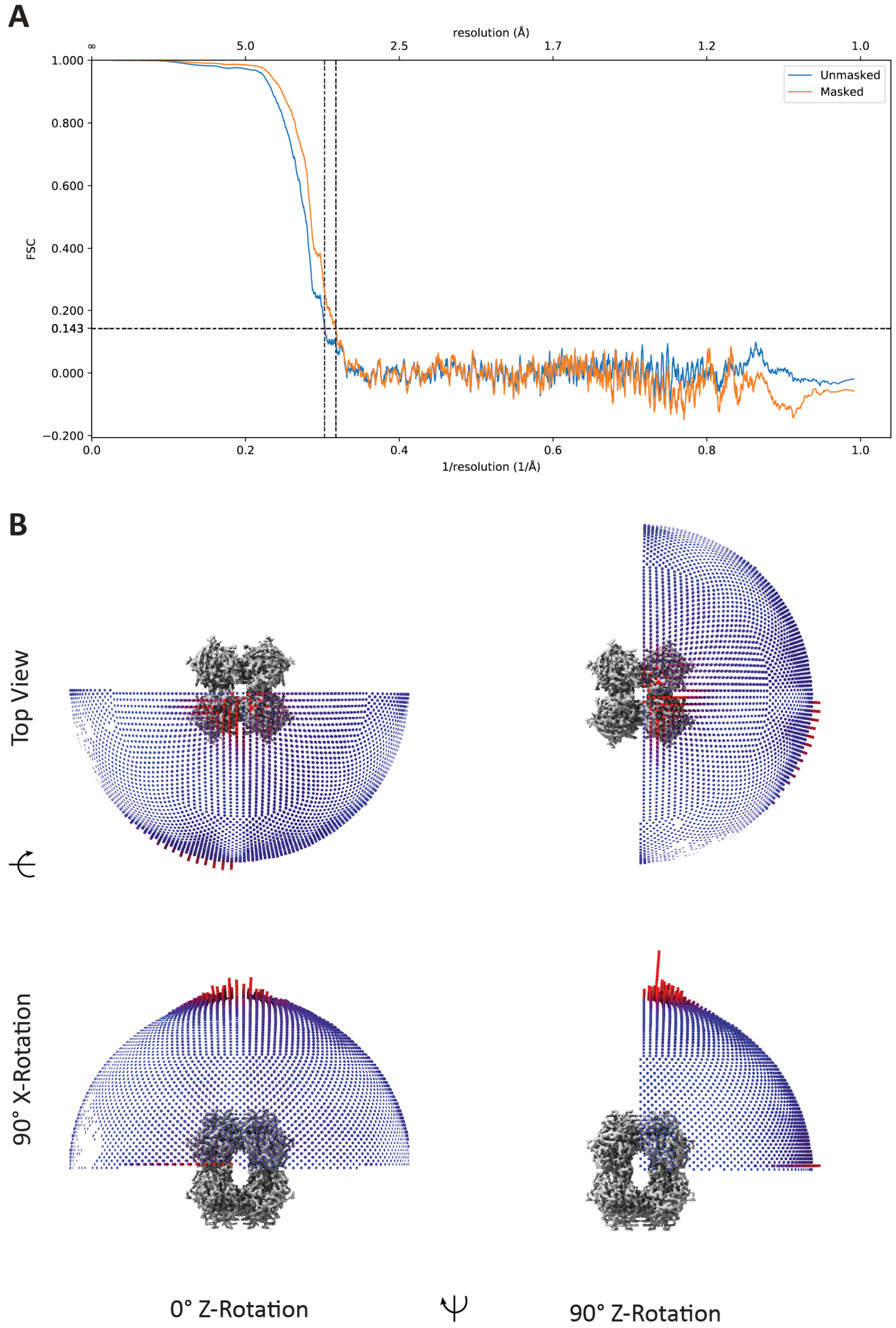

Supplementary Figure 2. Resolution and angular distribution of MFAP4 with Ca^{2+}. (**A**) FSC of the MFAP4 structure in the presence of Ca^{2+}. Resolution was calculated from the correlation between two independently refined halves of the data. Resolution at the FSC=0.143 criterion is 3.55 Å without masking and 3.15 Å with masking. (**B**) A 3D representation of the angular distribution of particles used in the MFAP4 structure with Ca^{2+}. Source data are provided as a Source Data file.

Supplementary Figure 3

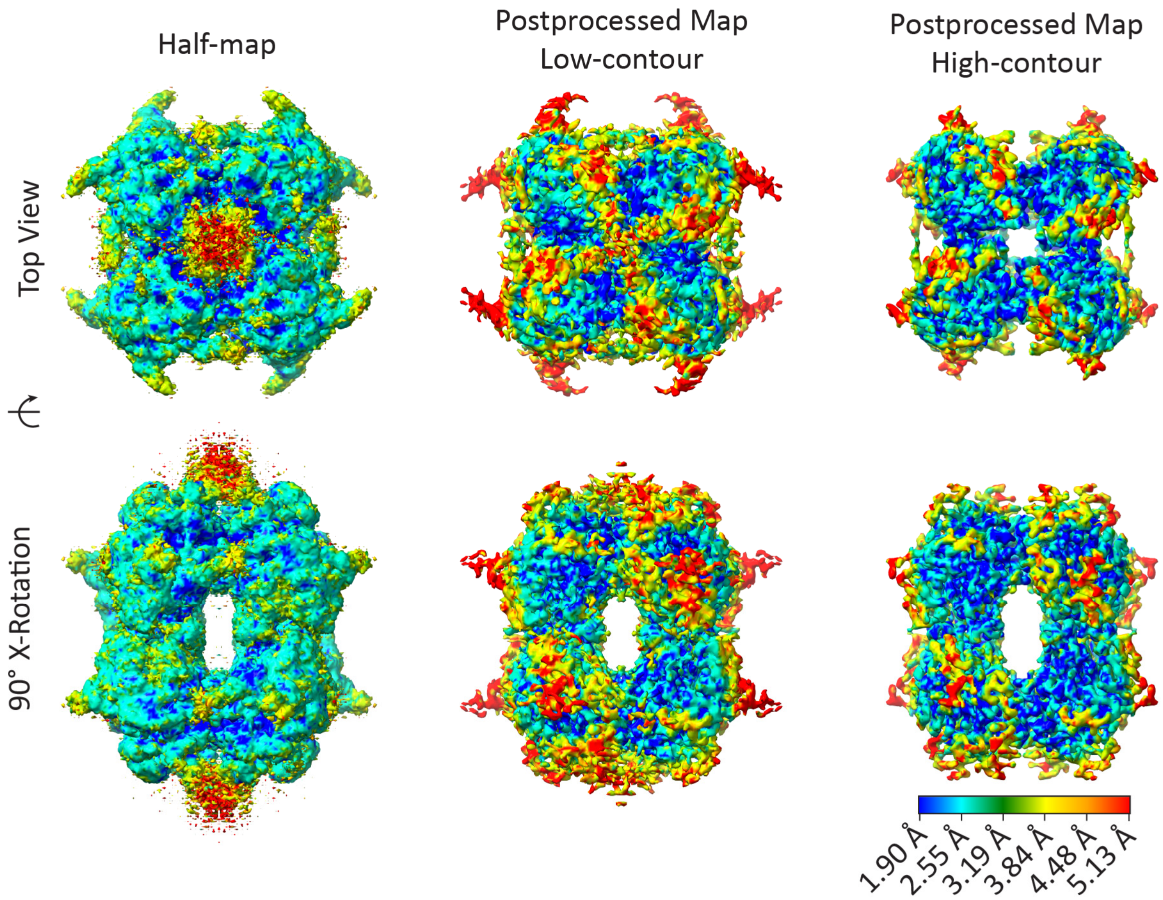

Supplementary Figure 3. Local resolution of MFAP4 with Ca^{2+}. Local resolution was calculated using Resmap (v1.1.4) [38] from one of the two independently refined half-maps. This local resolution estimate was projected onto the volume rendering of one of the half-maps as well as the B-factor sharpened post-processed map at low and high contour levels.

Supplementary Figure 4

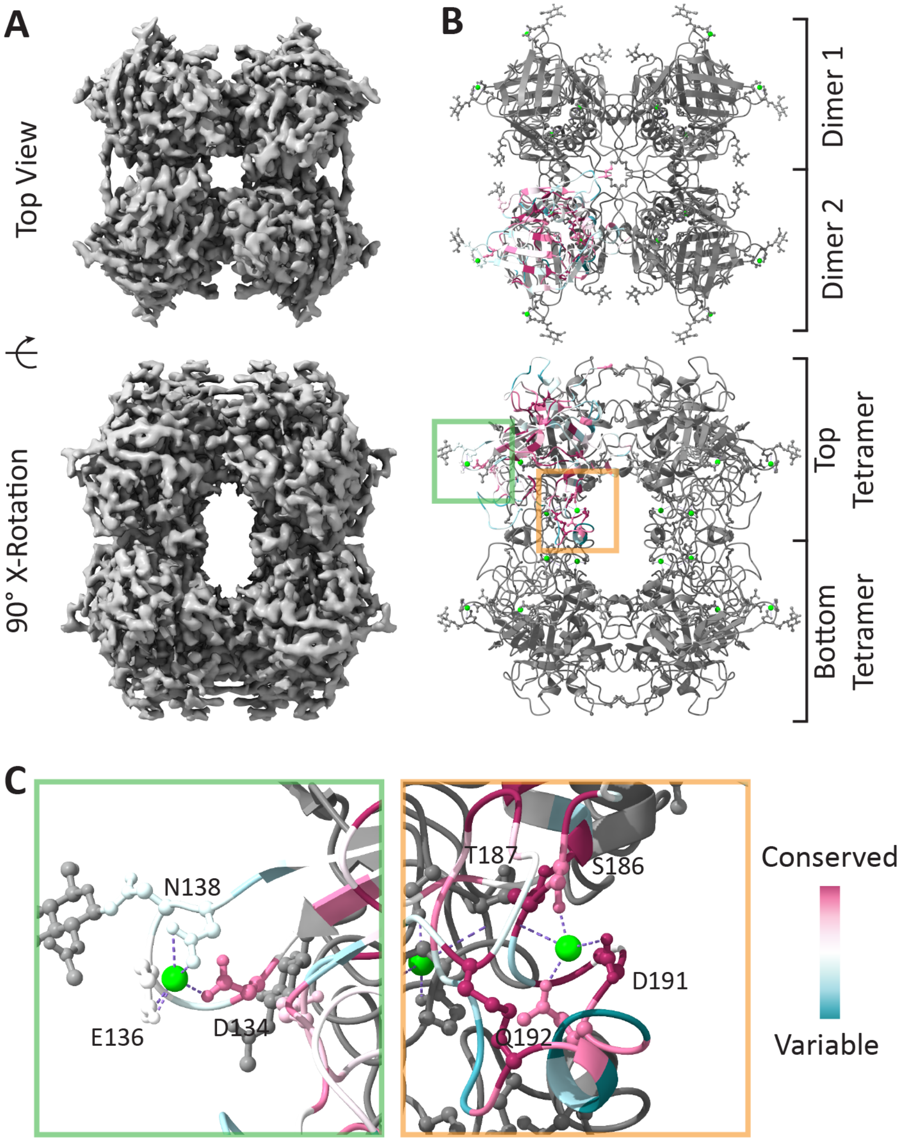

Supplementary Figure 4. Conservation of Ca^{2+} binding sites. (**A**) Top and side views of the 3.55 Å resolution cryo-EM map of MFAP4 with Ca^{2+} and (**B**) the atomic model colour coded using ConSurf [36]. Colour coding indicates conserved (magenta) to variable (blue) residues. (**C**) Close-up views of Ca^{2+}-binding sites highlighted in B show Ca^{2+}-binding near the glycosylated N137 (green square) and near the top/bottom tetramer interface (orange square).

Supplementary Figure 5

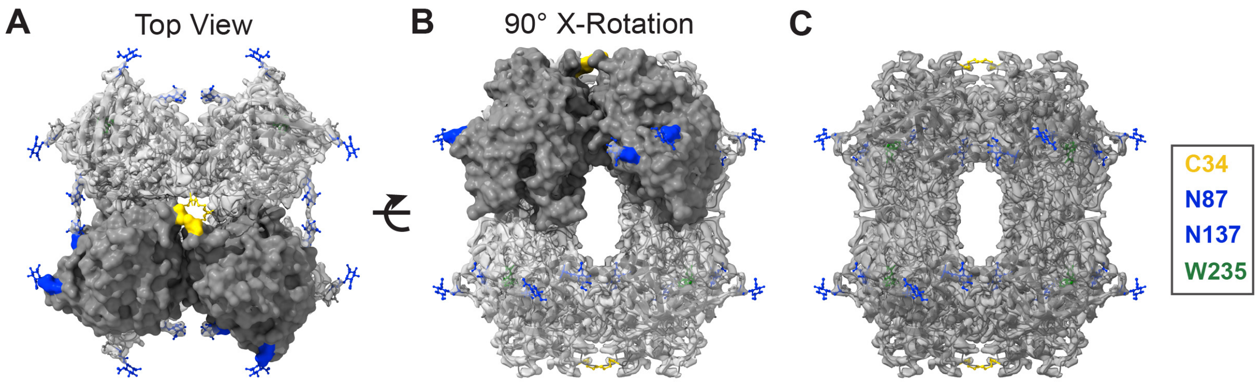

Supplementary Figure 5. Glycosylation of MFAP4 N87 and N137. (**A**) Top view of the cryo-EM map of MFAP4 with Ca^{2+} with superimposed ribbon model. Two disulfide-linked protomers are depicted with surface rendering. (**B**) Sideview with surface rendering and (**C**) without surface rendering. Intermolecular disulfide-bonded C34 (yellow), N87, and N137 with NAG glycans (blue), and W235 (green) atoms are shown.

Supplementary Figure 6

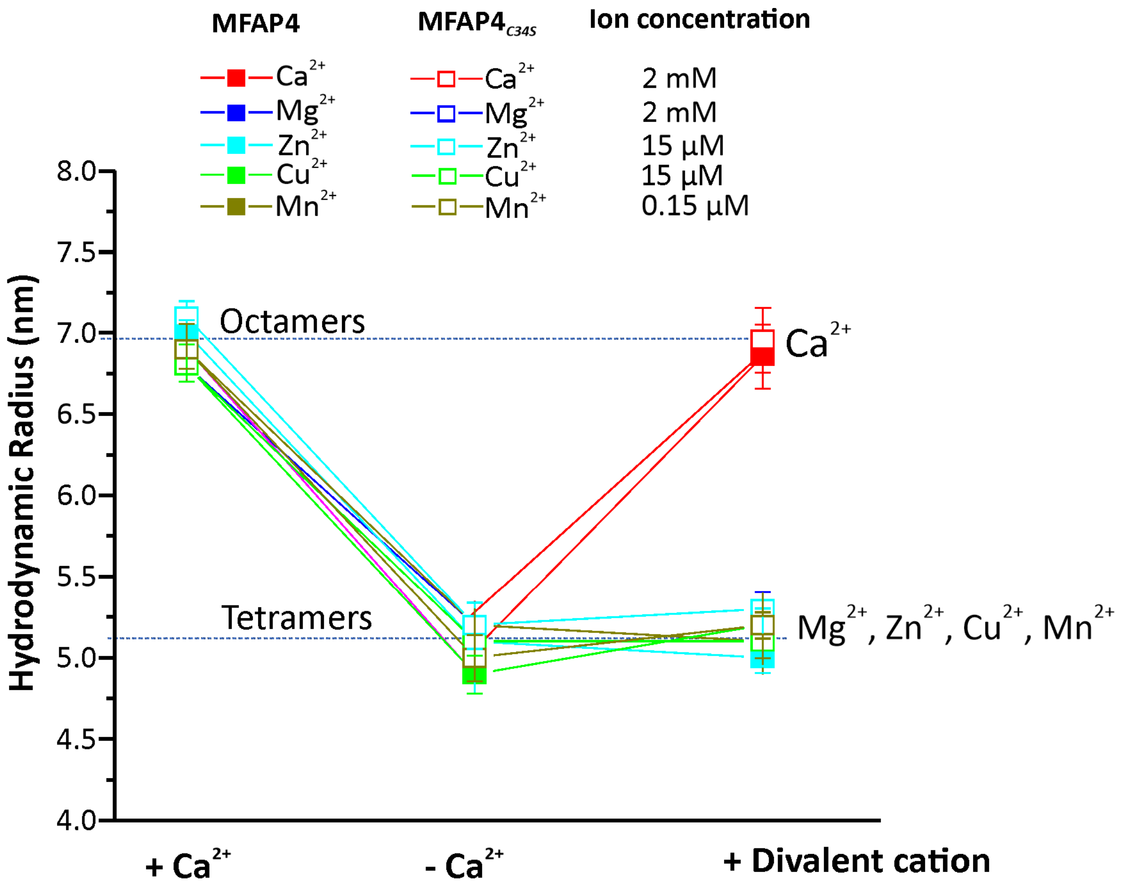

Supplementary Figure 6. **The MFAP4 octameric structure requires Ca^{2+} but not other divalent cations.** DLS analyses of Ca^{2}-depleted (5 mM EGTA for 20 min) MFAP4 and MFAP4$_{C34S}$ supplemented with either Ca^{2+}, Mg^{2+}, Zn^{2+}, Cu^{2+}, or Mn^{2+} at concentrations of physiological relevance as indicated. Data points are mean values of hydrodynamic radii derived from n=3 independent experiments each measured in technical triplicates. Error bars represent standard errors of the mean. Source data are provided as a Source Data file.

Supplementary Figure 7

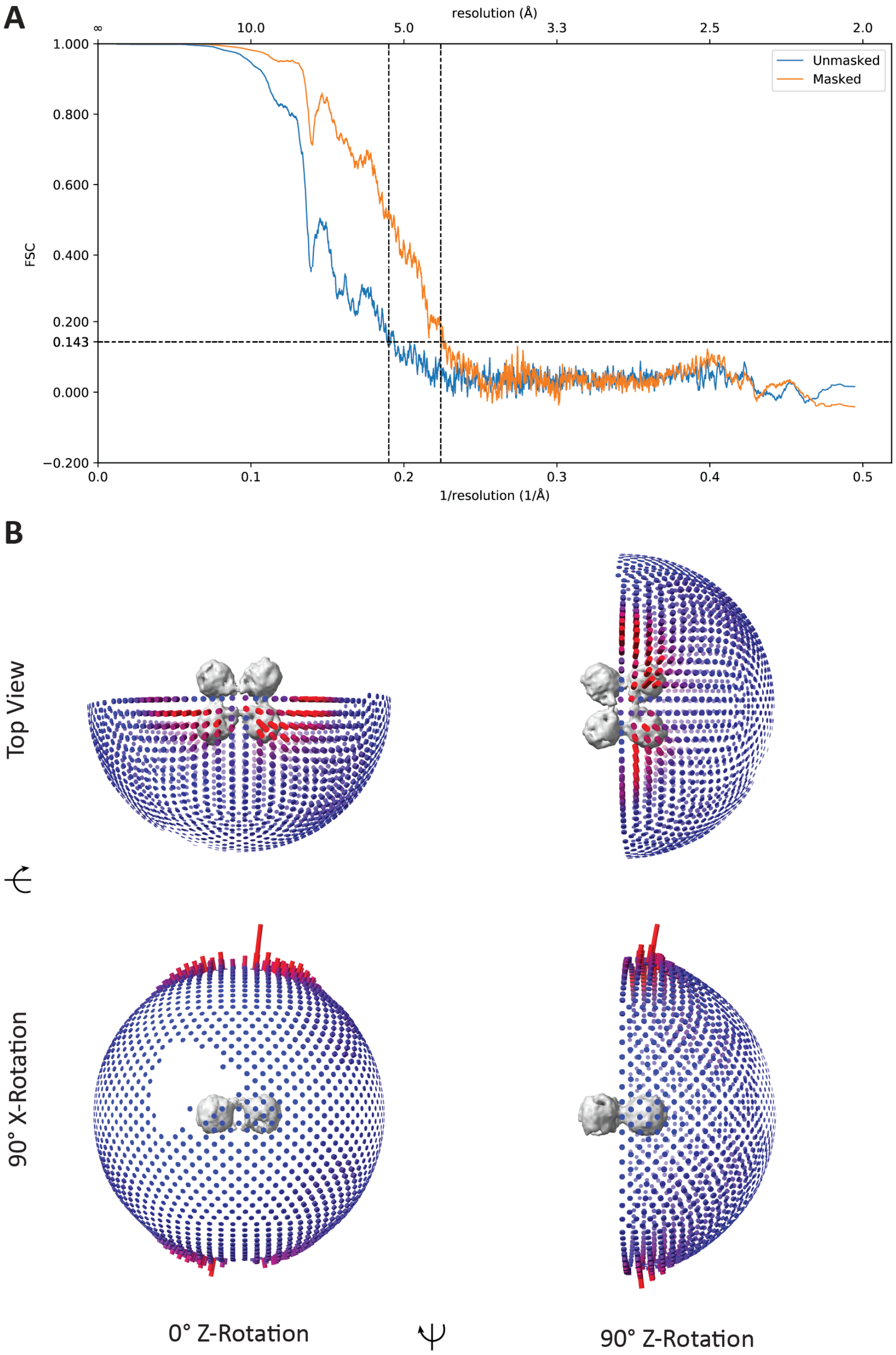

Supplementary Figure 7. Resolution and angular distribution of MFAP4 without Ca^{2+}. (**A**) FSC of the MFAP4 structure without Ca^{2+}. Resolution was calculated from the correlation between two independently refined halves of the data. Resolution at the FSC=0.143 criterion is 5.26 Å without masking and 4.46 Å with masking. (**B**) A 3D representation of the angular distribution of particles used in the MFAP4 structure without Ca^{2+}. Source data are provided as a Source Data file.

Supplementary Figure 8

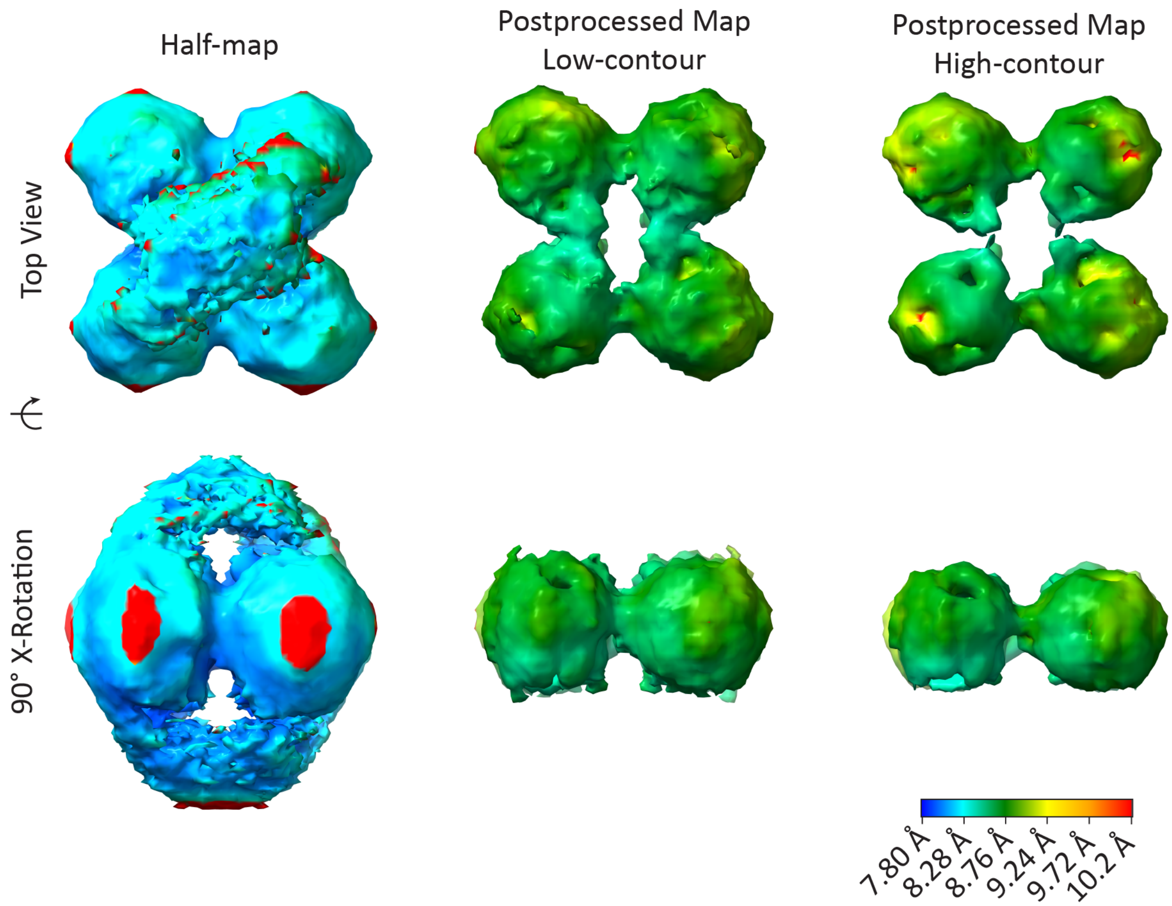

Supplementary Figure 8. Local resolution of MFAP4 without Ca^{2+}. Local resolution was estimated using CryoRes[39] from either one of the two independently refined half-maps or the B-factor sharpened postprocessed map. These local resolution maps were projected onto the respective volumes from which they were generated, either the half-map or post-processed map. Volume renderings of one of the half-maps as well as the B-factor sharpened postprocessed map are shown at low and high contour levels (centre and right panels).

Supplementary Figure 9

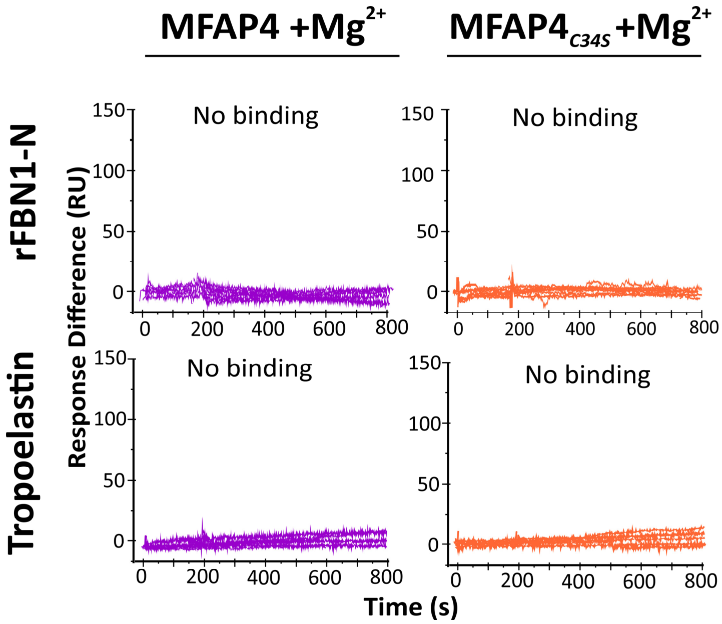

Supplementary Figure 9. MFAP4 and MFAP4*C34S* do not interact with fibrillin-1 and tropoelastin in the presence of Mg^{2+}. SPR analyses of the soluble analytes rFBN1-N (N-terminal half of fibrillin-1) and tropoelastin with MFAP4 and MFAP4*C34S* immobilized on the sensor surface in the presence of magnesium ions (+Mg^{2+}). Analyte concentrations were 100, 50, 20, 10, 5, and 0 µg/mL. Source data are provided as a Source Data file.

Supplementary Figure 10

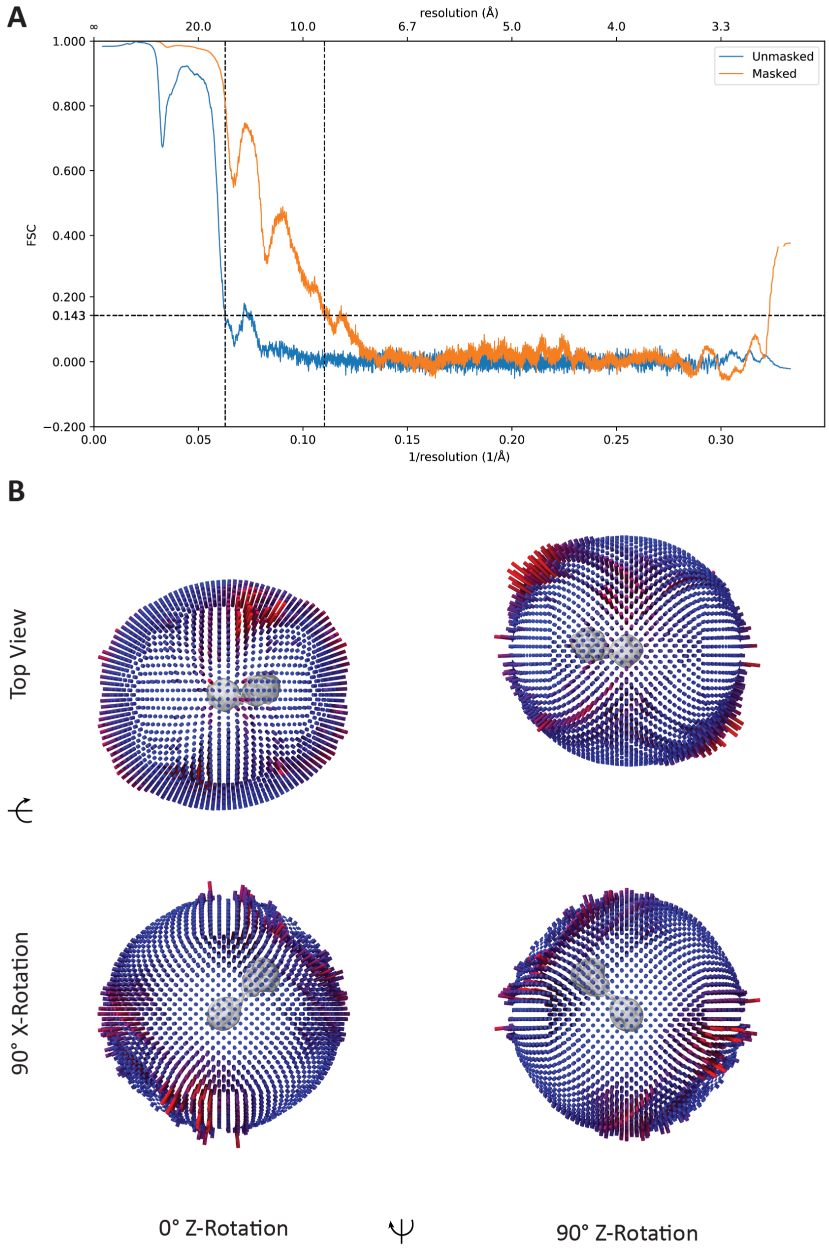

Supplementary Figure 10. Resolution and angular distribution of the MFAP4 octamer chain. (**A**) FSC of the MFAP4 octamer chain map. Resolution was calculated from the correlation between two independently refined halves of the data. Resolution at the FSC=0.143 criterion is 15.92 Å without masking and 9.07 Å with masking. (**B**) A 3D representation of the angular distribution of particles used in the MFAP4 octamer chain structure. Source data are provided as a Source Data file.

Supplementary Figure 11

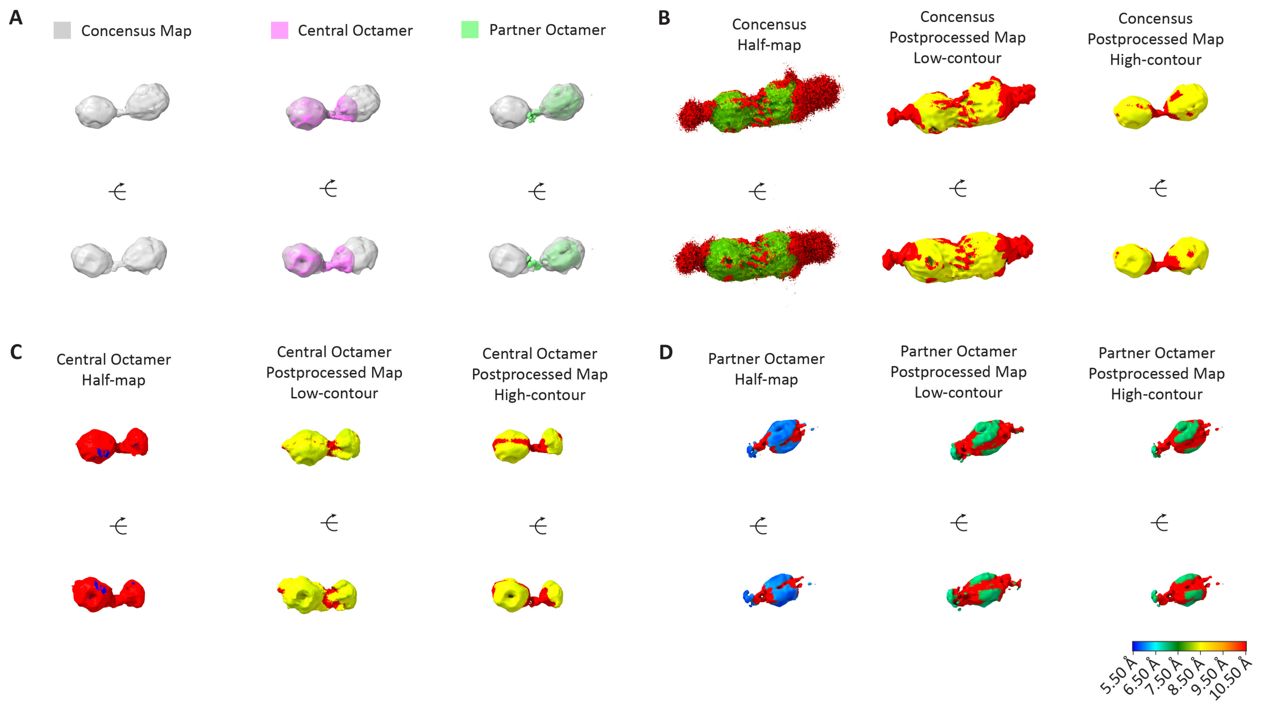

Supplementary Figure 11. Local resolution of MFAP4 octamer chain maps. Local resolution was estimated using CryoRes[39] from either one of the two independently refined half-maps or the B-factor sharpened postprocessed map. These local resolution maps were projected onto the respective volumes from which they were generated. Volume renderings of one of the half-maps as well as the B-factor sharpened postprocessed map are shown at low and high contour levels. (**A**) Depiction of the relative configuration of the central and partner octamers relative to the consensus map. Local resolution maps are shown in (**B**) for the consensus map, as well as for the multibody refined central octamer map in (**C**) and the partner octamer map in (**D**).

Supplementary Figure 12

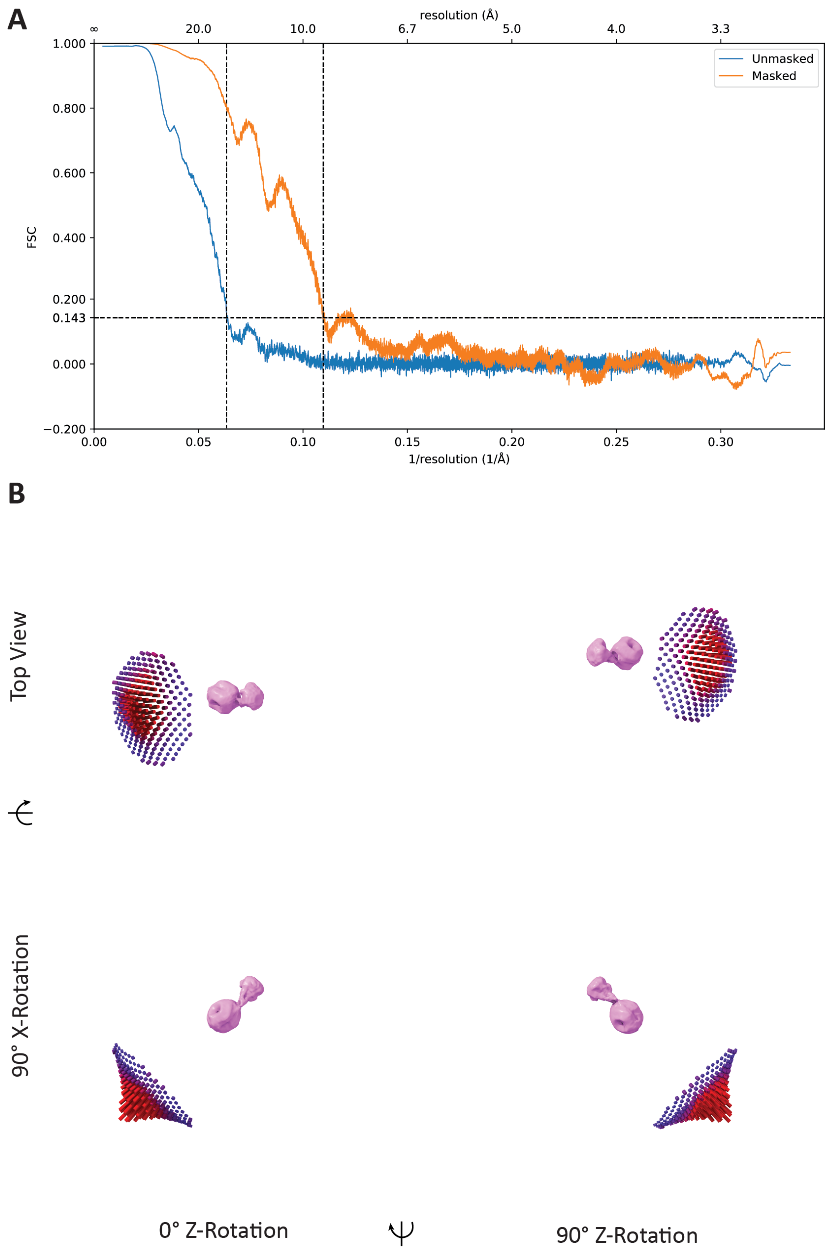

Supplementary Figure 12. Resolution and angular distribution of the MFAP4 octamer chain's central octamer. (**A**) FSC of the central octamer following multibody refinement of the MFAP4 octamer chain. Resolution was calculated from the correlation between two independently refined halves of the data. Resolution at the FSC=0.143 criterion is 15.77 Å without masking and 9.13 Å with masking. (**B**) A 3D representation of the angular distribution of particles used in the MFAP4 octamer chain structure. Source data are provided as a Source Data file.

Supplementary Figure 13

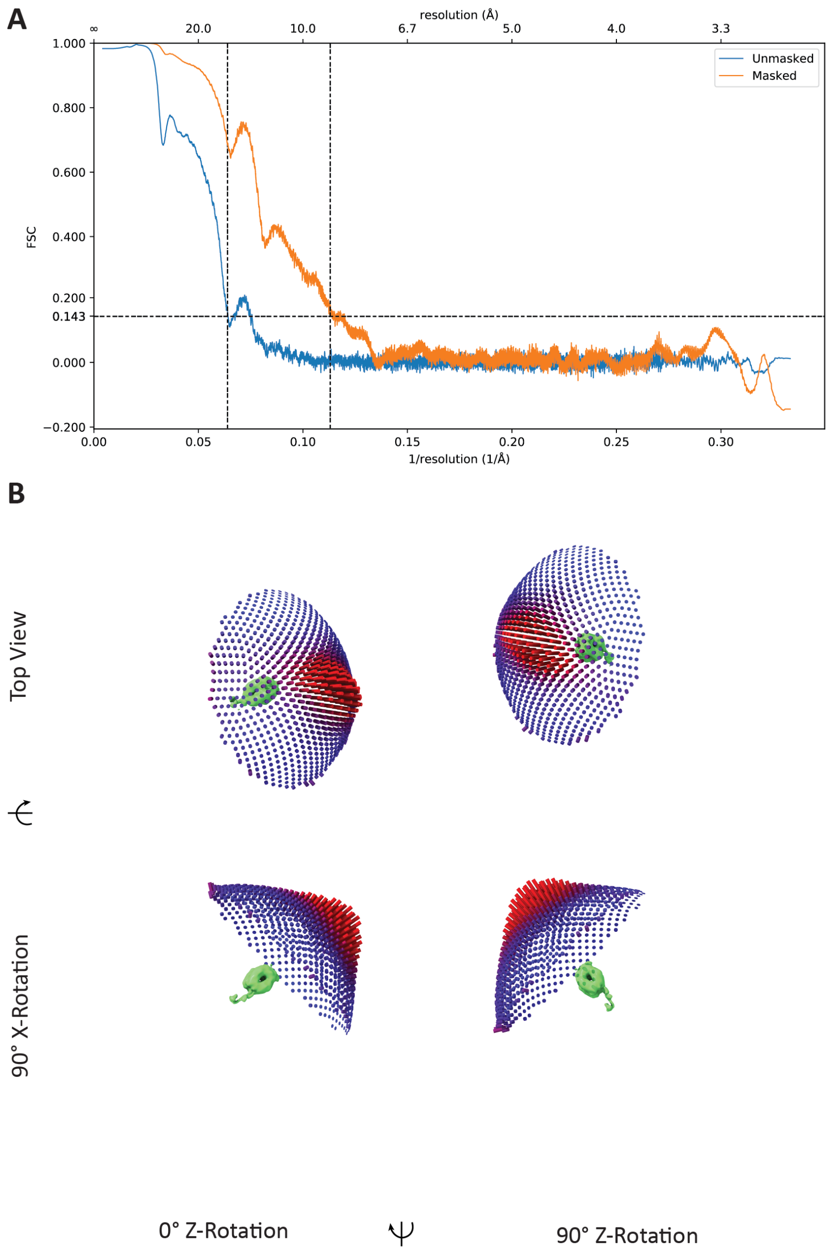

Supplementary Figure 13. Resolution and angular distribution of the MFAP4 octamer chain's partner octamer. (**A**) FSC of the partner octamer following multibody refinement of the MFAP4 octamer chain. Resolution was calculated from the correlation between two independently refined halves of the data. Resolution at the FSC=0.143 criterion is 15.60 Å without masking and 8.84 Å with masking. (**B**) A 3D representation of the angular distribution of particles used in the MFAP4 octamer chain structure. Source data are provided as a Source Data file.

Supplementary Figure 14

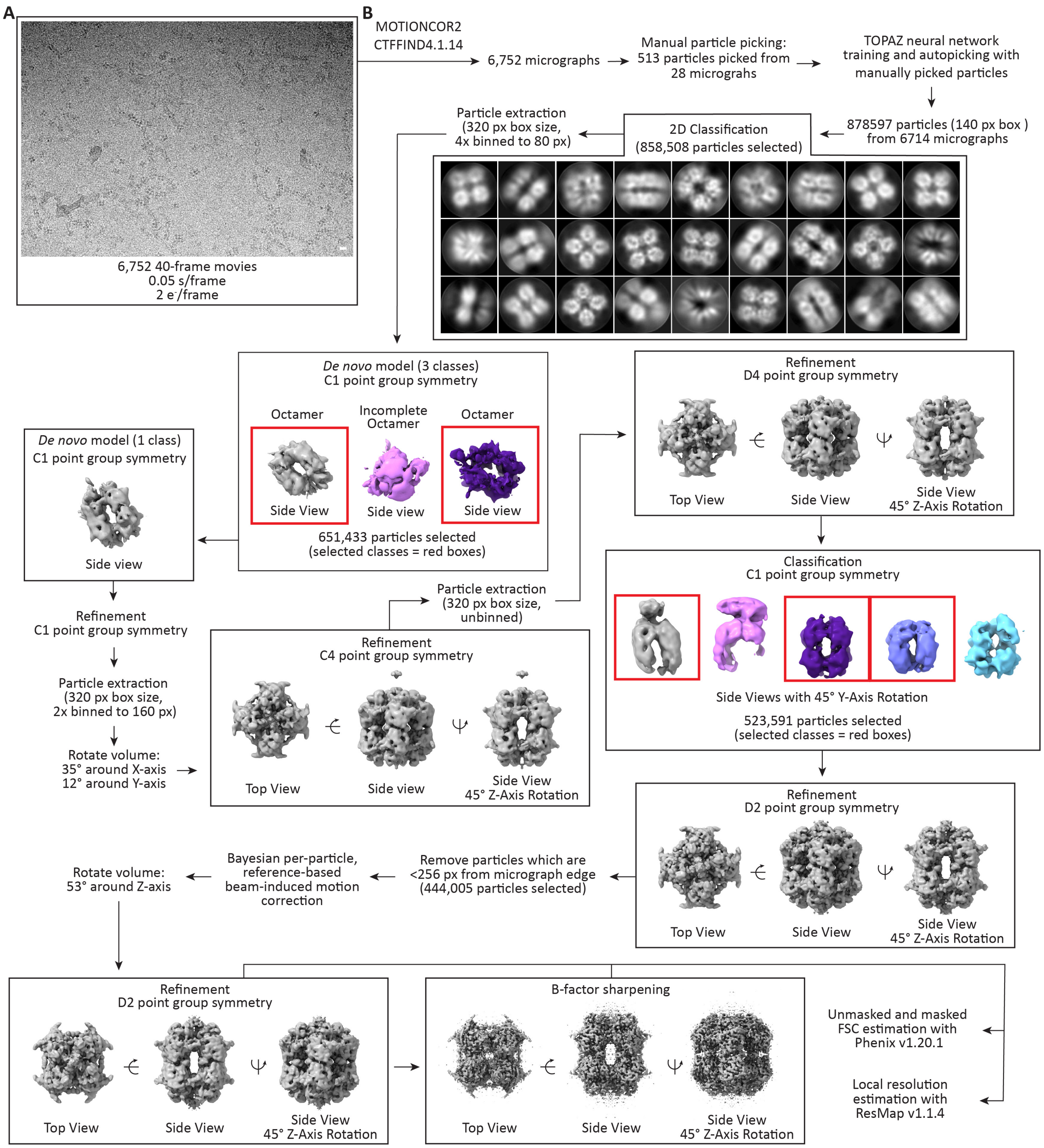

Supplementary Figure 14. Single particle analysis workflow overview of MFAP4 with Ca^{2+}. (**A**) A representative micrograph from a 40-frame, 2 s total exposure movie following (**B**) motion-correction with MotionCor2 [48] (10 nm scale bar). CTF was estimated with CTFFIND4.1.14 [49]. Manually picked coordinates were used to train a Topaz model for automatic particle picking [58]. Selected 2D classes were used as input for *de novo* initial reference generation with C1 point group symmetry. Reference maps were manually rotated with Sparx [50] first to align the MFAP4 assembly along the Z-axis and later to position dimer units within each half defined by the two-fold axis of the D2 structure. The D2 point group was applied during final refinement. Bayesian per-particle beam-induced motion correction, as well as B-factor sharpening, was performed with RELION4-beta [40]. FSC was estimated with Phenix v1.20.1 [34]. Local resolution was estimated with ResMap v1.1.4 [38].

Supplementary Figure 15

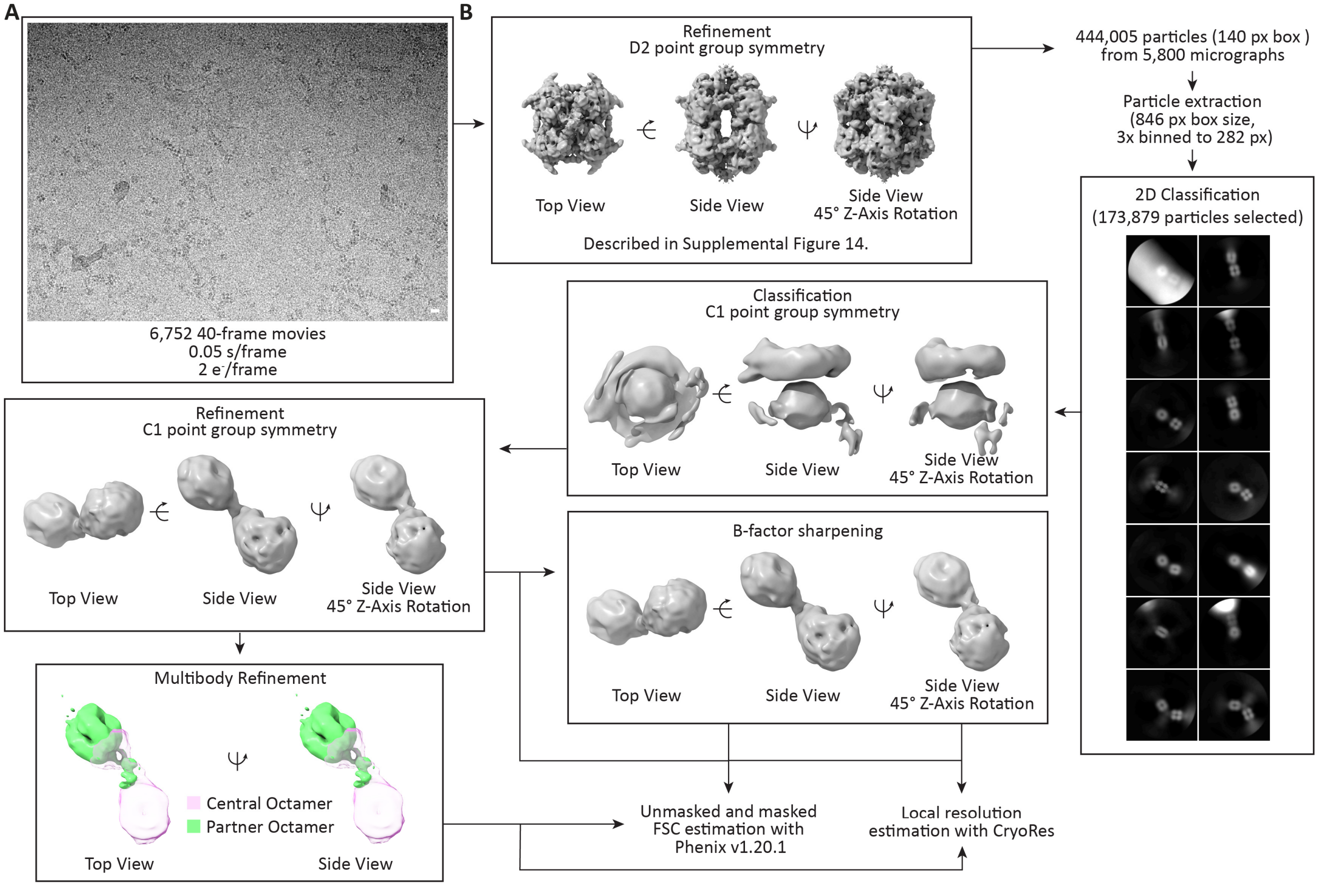

Supplementary Figure 15. Single particle analysis workflow overview of MFAP4 chain with Ca^{2+}. (**A**) A representative micrograph from a 40-frame, 2 s total exposure movie following motion-correction with MotionCor2 [48] and CTF was estimated with CTFFIND4.1.14 [49] (10 nm scale bar). Refined coordinates of MFAP4 with Ca^{2+} (described in Supplementary Figure 14) were used for extraction and 2D classification. Selected 2D classes were used as input for classification with C1 point group symmetry to produce a single class reference for subsequent refinement. Refined C1 point group symmetry coordinates and references were segmented and masked to create separate central (pink) and partner (green) octamer reference maps for multibody refinement. Maps were B-factor sharpened with RELION4-beta. FSC was estimated with Phenix v1.20.1 [34]. Local resolution was estimated with CryoRes [39]

Supplementary Figure 16

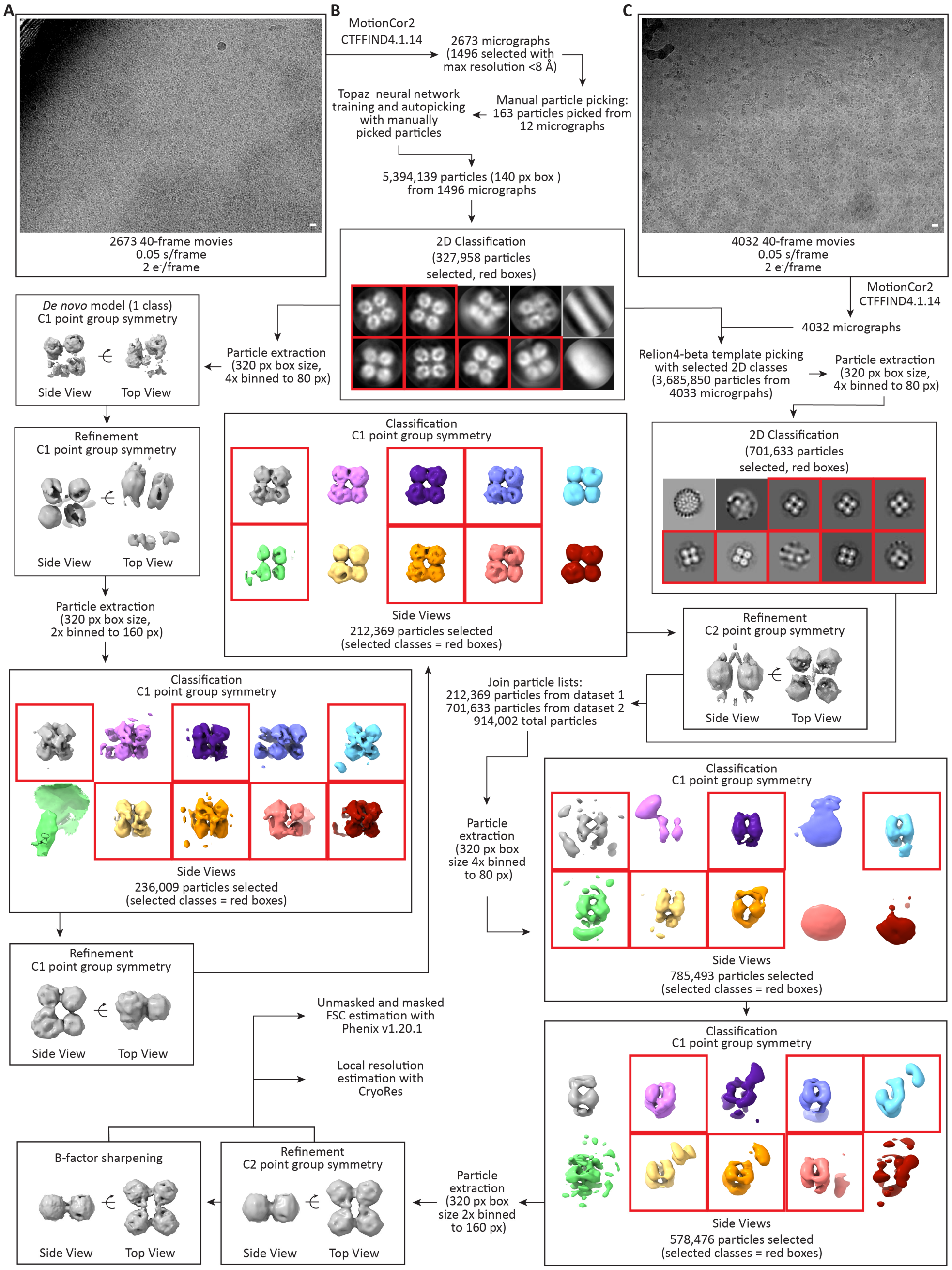

Supplementary Figure 16. Single particle analysis workflow overview of MFAP4 without Ca^{2+}. (**A**) A representative micrograph from a 40-frame, 2 s total exposure movie following (**B**) motion-correction with MotionCor2 [48] (dataset 1). CTF was estimated with CTFFIND4.1.14 [49]. Manually picked coordinates were used to train a Topaz model for automatic particle picking [58]. Selected 2D classes were used as input for *de novo* initial reference generation. Iterative 3D refinement and classification were used to refine references with C1 point group symmetry. C2 point group symmetry 3D refinement produced a reference with four spherical bodies; occupying separate quadrants drawn within the XY-plane. (C) Additional movies were collected of MFAP4 without Ca^{2+} (dataset 2) with the same microscope and imaging parameters as dataset 1 (10 nm scale bar). 2D classes from dataset 1 were used for template matching with RELION4-beta [40]. Dataset 2 coordinates from template matching were combined with the coordinates of the C2 point group symmetry reference from dataset 1. Classification was used to select particles for extraction, further refinement with C2 point group symmetry and B-factor sharpening with RELION4-beta. FSC was estimated with Phenix v1.20.1 [34]. Local resolution was estimated with CryoRes [39].

Supplementary Figure 17

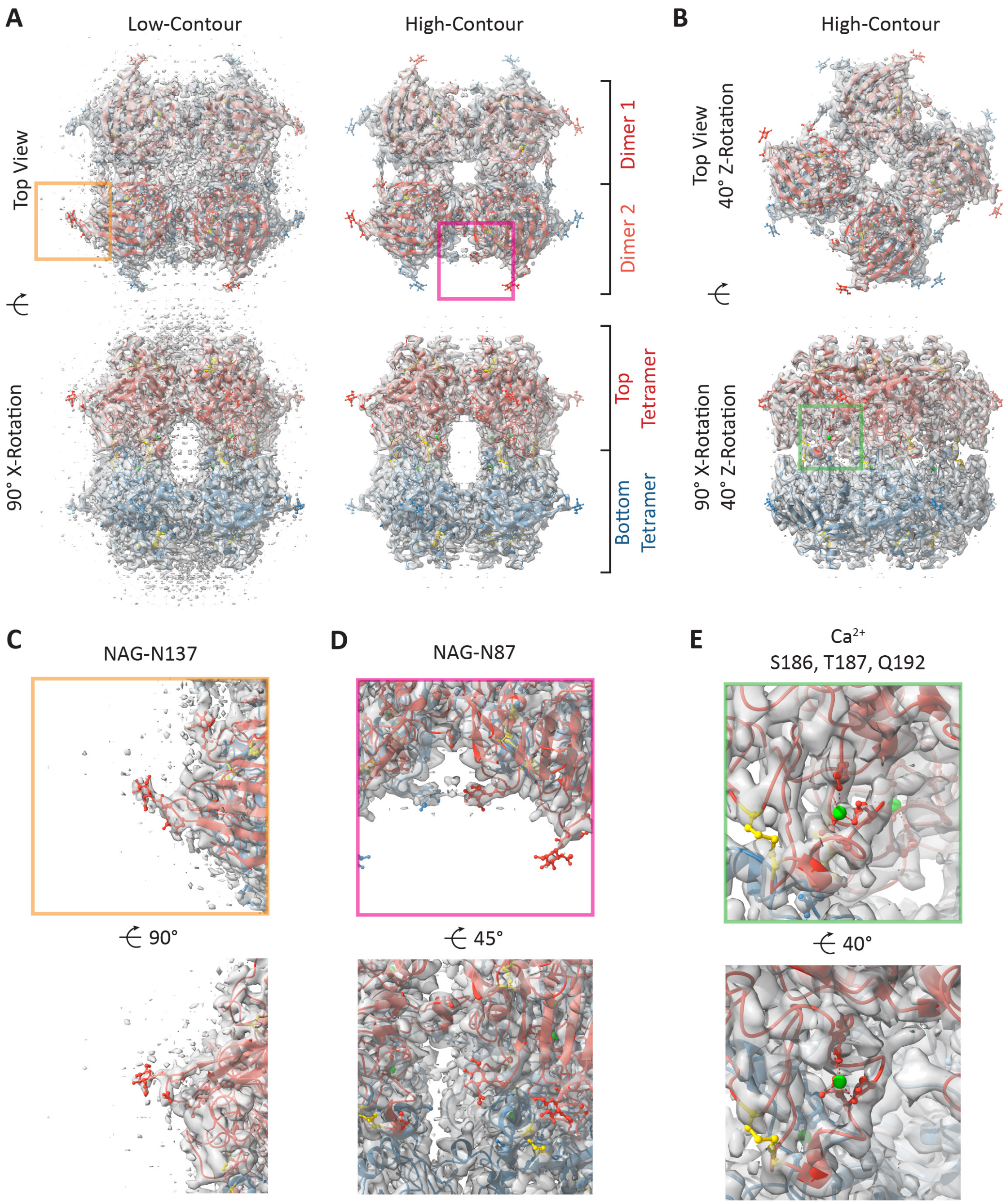

Supplementary Figure 17. Ligands NAG-N137, NAG-N87, and Ca^{2+}-S186, T187, Q192 within the cryo-EM density map. (**A**) Atomic model and cryo-EM density map of MFAP4 with Ca^{2+} displayed as low- and high-contour levels with ligands NAG-N137, NAG-N87, and Ca^{2+}-S186, T187, Q192 displayed as ball-and-stick models. (**B**) Model and map 40° rotated around the Z-axis. (**C**) Close-up view of the NAG-N137 within the orange box of panel A. (**D**) Close-up view of the NAG-N87 within the magenta box of panel A. (**E**) Close-up view of the Ca^{2+}-S186, T187, Q192 within the green box of panel B (Ca^{2+} ions are depicted in green).

Supplementary Table 1

MFAP4 interactions with elastogenic full-length and sub-fragment proteins in the presence of Ca^{2+} (octameric form) determined by SPR. This summary table includes in addition to the values shown in Figs. 3 and 4 other tested interactions.

**Binding Ligand**	**K_D (nM)**	**Binding Strength**	**Ca^{2+}-Dependency**
MFAP4 (self-interaction)	6.2 ± 0.9	Very strong	Yes
rFBN1-N (Fibrillin-1, N-terminal half)	1.8 ± 0.9	Very strong	Yes
rFBN1-C (Fibrillin-1, C-terminal half)	No binding	No binding	Not applicable
rF1M (Fibrillin-1, centre region)	1.2 ± 0.5	Very strong	Yes
Tropoelastin	45 ± 9	Strong	Yes
LTBP4L	18 ± 5	Strong	No
LTBP4S	15 ± 6	Strong	No
LTBP4L (N-terminal half)	19 ± 6	Strong	Not determined
LTBP4S (N-terminal half)	16 ± 4	Strong	Not determined
LTBP4L/S (C-terminal half)	No binding	No binding	Not applicable
Fibulin-3	No binding	No binding	Not applicable
Fibulin-4	110 ± 33	Moderate	Yes
Fibulin-5	357 ± 47	Moderate	Yes
Fibronectin	No binding	No binding	Not applicable

Supplementary Table 2

Double-stranded synthetic DNA sequence and oligonucleotides used to generate the expression plasmids for MFAP4 and MFAP4$_{C34S}$.

**Name**	**DNA sequence (5' – 3')**
gBlock human MFAP4	gcagagctcgtttagtgaaccgtcagatctctagaagctgggtaccagctgctagcgctg<br>agcatgaaggcactcctggccctgccgctgctgctgcttctctccacgcccccgtgtgcc<br>ccccaggtctccgggatccgaggagatgctctggagaggttttgccttcagcaacccctg<br>gactgtgacgacatctatgcccagggctaccagtcagacggcgtgtacctcatctacccc<br>tcgggccccagtgtgcctgtgcccgtcttctgtgacatgaccaccgagggcgggaagtgg<br>acggttttccagaagagattcaatggctcagtaagtttcttccgcggctggaatgactac<br>aagctgggcttcggccgtgctgatggagagtactggctggggctgcagaacatgcacctc<br>ctgacactgaagcagaagtatgagctgcgagtggacttggaggactttgagaacaacacg<br>gcctatgccaagtacgctgacttctccatctccccgaacgcggtcagcgcagaggaggat<br>ggctacaccctctttgtggcaggctttgaggatggcggggcaggtgactccctgtcctac<br>cacagtggccagaagttctctaccttcgaccgggaccaggacctctttgtgcagaactgc<br>gcagctctctcctcaggagccttctggttccgcagctgccactttgccaacctcaatggc<br>ttctacctaggtggctcccacctctcttatgccaatggcatcaactgggcccagtggaag<br>ggcttctactactccctcaaacgcactgagatgaaaatccgccgggccggtaagcctatc<br>cctaaccctctcctcggtctcgattctacccgtaccggtcatcaccatcaccatcaccat<br>cactgataagcggccgctcgaggccggcaaggccggatccagacatgataagatacattg<br>atgagt
Oligo 1 MFAP4$_{C34S}$	gatgctctggagaggttt**gc**ccttcagcaacccct
Oligo 2 MFAP4$_{C34S}$	aggggttgctgaagg**gc**aaacctctccagagcatc

Supplementary Table 3

Cryo-EM data collection, refinement, and validation statistics

	#1 MFAP4 +Ca^{2+} (EMDB-42394) (PDB 8UN7)	#2 MFAP4 -Ca^{2+} (EMDB-42398) (PDB *N/A*)
**Data collection and processing**		
Magnification	105 kx	105 kx
Voltage (kV)	300 kV	300 kV
Electron exposure (e–/Å^2)	80	80
Defocus range (μm)	1.5 to 3.5	1.5 to 3.5
Pixel size (Å)	0.8550	0.8550
Symmetry imposed	D2	C2
Initial particle images (no.)	878,597	914,002
Final particle images (no.)	444,005	578,476
Map resolution (Å)	3.55	5.26
FSC threshold	0.143	0.143
Map resolution range (Å)	1.90 to 5.13	5.26 to 10.20
**Refinement**		
Initial model used (PDB code)	Supplemental Data File 1	
Model resolution (Å)	3.24	
FSC threshold	0.143	
Model resolution range (Å)	1.90 to 5.13	
Map sharpening *B* factor (Å^2)	144.915	
Model composition		
Non-hydrogen atoms	14480	
Protein residues	1776	
Ligands	NAG:16<br>Ca:8	
B factors (Å^2)	Min/Max/Mean	
Protein	0.00/144.92/54.99	
Ligand	69.39/116.52/93.10	
R.m.s. deviations		
Bond lengths (Å)	0.002	
Bond angles (°)	0.553	
Validation		
MolProbity score	1.91	
Clashscore	5.21	
Poor rotamers (%)	0.27	
Ramachandran plot		
Favored (%)	86.59	
Allowed (%)	12.73	
Disallowed (%)	0.68	
