# Supplementary material for: Microfibril-associated glycoprotein 4 forms octamers that mediate interactions with elastogenic proteins and cells
Source: Nat Commun. 2024 May 13;15:4015. doi: 10.1038/s41467-024-48377-z (PMC11091212; doi:10.1038/s41467-024-48377-z)
Supplement: Supplementary file 3 — Description of Additional Supplementary Files [file 41467_2024_48377_MOESM3_ESM.pdf]

Description of Additional Supplementary Files

File Name: Supplementary Data 1
Description: Structural model of human MFAP4 predicted with AlphaFold Colab.

File Name: Supplementary Movie 1
Description: Cryo-EM map and integrative atomic model of MFAP4 with Ca^{2+}. D2 point group symmetry 3D cryo-EM map of MFAP4 along with superimposed integrative atomic model (Q37-A255 resolved by cryo-EM map, intermolecular disulfide bonds between C34 interpreted from presented biochemical and mutagenesis evidence, positions of C34-Q36 were modelled to minimise deviation from the expected bond length and angle restrictions in Coot 0.9.8.8). Intermolecular disulfide bonded protomer pairs are colour coded accordingly to be the same shade of red or sky-blue. Cysteine residues are coloured yellow. Four N-termini (residues 21-34) of superimposed AlphaFold model is briefly shown aligned to protomers within the presented atomic model; the N-terminal RGD motifs of these AlphaFold models are highlighted (orange). N87 and N137 along with NAG glycans are shown (blue).

File Name: Supplementary Movie 2
Description: Intra-tetrameric interactions of MFAP4 with Ca^{2+}. Intra-tetrameric interactions within the octamer assembly coloured according to inter-chain distance (dark orange <2.5 Å, light orange <8.5 Å; see colour scale in Fig. 2F/G). Cysteine residues are coloured yellow in all subpanels. Hydrogen bonds are depicted as dashed blue lines. Ca^{2+} ions are depicted as green spheres.

File Name: Supplementary Movie 3.
Description: Inter-tetrameric interactions of MFAP4 with Ca^{2+}. Inter-tetrameric interactions within the octamer assembly coloured according to inter-chain distance (dark orange <2.5 Å, light orange <8.5 Å; see colour scale in Fig. 2F/G). Cysteine residues are coloured yellow in all subpanels. Hydrogen bonds are depicted as dashed blue lines. Ca^{2+} ions are depicted as green spheres.

File Name: Supplementary Movie 4.
Description: Central and partner octamers within MFAP4 chain-like assemblies. Rotated movie of the consensus map (grey) of the central (pink) and partner (green) MFAP4 octamers in TBS/Ca^{2+} following multibody refinement. The first three principal components of movement are also shown, corresponding to the first (red) and second (yellow) swinging movement and the third twisting movement (blue) of the partner relative to the central MFAP4.
