# Supplementary material for: Microfibril-associated glycoprotein 4 forms octamers that mediate interactions with elastogenic proteins and cells
Source: Nat Commun. 2024 May 13;15:4015. doi: 10.1038/s41467-024-48377-z (PMC11091212; doi:10.1038/s41467-024-48377-z)
Supplement: Supplementary file 9 — Reporting Summary [file 41467_2024_48377_MOESM9_ESM.pdf]

nature portfolio

Corresponding author(s): Dieter P. Reinhardt

Last updated by author(s): Apr 18, 2024

Reporting Summary

Nature Portfolio wishes to improve the reproducibility of the work that we publish. This form provides structure for consistency and transparency in reporting. For further information on Nature Portfolio policies, see our Editorial Policies and the Editorial Policy Checklist.

Statistics

For all statistical analyses, confirm that the following items are present in the figure legend, table legend, main text, or Methods section.

n/a	Confirmed	
☐	☒	The exact sample size (n) for each experimental group/condition, given as a discrete number and unit of measurement
☐	☒	A statement on whether measurements were taken from distinct samples or whether the same sample was measured repeatedly
☐	☒	The statistical test(s) used AND whether they are one- or two-sided<br>*Only common tests should be described solely by name; describe more complex techniques in the Methods section.*
☒	☐	A description of all covariates tested
☐	☒	A description of any assumptions or corrections, such as tests of normality and adjustment for multiple comparisons
☐	☒	A full description of the statistical parameters including central tendency (e.g. means) or other basic estimates (e.g. regression coefficient) AND variation (e.g. standard deviation) or associated estimates of uncertainty (e.g. confidence intervals)
☐	☒	For null hypothesis testing, the test statistic (e.g. F, t, r) with confidence intervals, effect sizes, degrees of freedom and P value noted<br>*Give P values as exact values whenever suitable.*
☒	☐	For Bayesian analysis, information on the choice of priors and Markov chain Monte Carlo settings
☒	☐	For hierarchical and complex designs, identification of the appropriate level for tests and full reporting of outcomes
☒	☐	Estimates of effect sizes (e.g. Cohen's d, Pearson's r), indicating how they were calculated

Our web collection on statistics for biologists contains articles on many of the points above.

Software and code

Policy information about availability of computer code

Data collection	DLS: Dynamics V6 (Dyna Pro/Wyatt). SPR: BIAControll (Bia core/ Cytiva) . AFM: NanoScope. TEM : Digital Micrograph. Cryo-TEM : SerialEM (https://bio3d.colorado.edu/SerialEM/).
Data analysis	DLS: Dynamics V6 (DynaPro/Wyatt).<br><br>SPR: BIAEvaluation (Biacore/Cytiva). SPR analysed data were first analysed and plotted with BIAEvaluation using correction of the bulk shifts at the start and stop of the analyte injection due to typical refractive index changes when the analyte enters and exits the sensor.<br><br>AFM: NanoScope.<br><br>Cell-attachment: ImageJ/Fiji (https://imagej.net/software/fiji/).<br><br>Cryo-EM Single-particle analysis and model building: Relion 4.0-beta (https://relion.readthedocs.io/en/release-4.0/Installation.html), AlphaFold Colab (https://colab.research.google.com/github/deepmind/alphafold/blob/main/notebooks/AlphaFold.i559 pynb), UCSF ChimeraX vl.6 (https://www.cgl.ucsf.edu/chimerax/), MotionCor2 (https://emcore.ucsf.edu/ucsf-software), CTFFIND4 (https://grigoriefflab.umassmed.edu/ctffind4), Sparx (https://cryoem.bcm.edu/downloads/view_eman2_versions), Coot v0.9.8.l (https://www2.mrc-lmb.cam.ac.uk/personal/pemsley/coot/), Phenix vl.20.1-4487 (https://phenix-online.org/), CryoRes (https://cryonet.ai/resolution/), custom script for residue distances between chains: pdb_at_dist_to_pdb_v2.py (https://zenodo.org/records/10833841), custom script to generate equally spaced points for cylindrical mask along vector between points: points_for_cylinder_mask.py (https://zenodo.org/records/10988630), and custom script to generate mask from model points: make_mask_from_model.py (https://zenodo.org/records/10834516).

Graphs of analysed data were generated with OriginPro 2023.

For manuscripts utilizing custom algorithms or software that are central to the research but not yet described in published literature, software must be made available to editors and reviewers. We strongly encourage code deposition in a community repository (e.g. GitHub). See the Nature Portfolio guidelines for submitting code & software for further information.

Data

Policy information about availability of data

All manuscripts must include a data availability statement. This statement should provide the following information, where applicable:

- Accession codes, unique identifiers, or web links for publicly available datasets
- A description of any restrictions on data availability
- For clinical datasets or third party data, please ensure that the statement adheres to our policy

Data generated in this study were deposited at the Electron Microscopy Data Bank (www.ebi.ac.uk/emdb; accession numbers EMD-42394, EMD-42398) and the Protein Data Bank (https://www.rcsb.org; accession number 8UN7). Custom scripts are available on zenodo (https://zenodo.org/records/10833841; https://zenodo.org/records/10988630; https://zenodo.org/records/10834516). All other data are available in the article and the supplemental data file. All source raw data are provided as a Source Data file.

Human research participants

Policy information about studies involving human research participants and Sex and Gender in Research.

Reporting on sex and gender	No human participants or data were used in this study.
Population characteristics	No human participants or data were used in this study.
Recruitment	No human participants or data were used in this study.
Ethics oversight	No human participants or data were used in this study.

Note that full information on the approval of the study protocol must also be provided in the manuscript.

Field-specific reporting

Please select the one below that is the best fit for your research. If you are not sure, read the appropriate sections before making your selection.

☒ Life sciences ☐ Behavioural & social sciences ☐ Ecological, evolutionary & environmental sciences

For a reference copy of the document with all sections, see nature.com/documents/nr-reporting-summary-flat.pdf

Life sciences study design

All studies must disclose on these points even when the disclosure is negative.

Sample size	The maximum available samples were used for data acquisition.
Data exclusions	Cryo-EM particles were excluded based on classification in Relion 4.0-beta and visual differences between classes related to quality of 2D classes or reconstructions of 3D classes. SPR association and dissociation binding curves were post-processed by excluding data at the start and stop of the analyte injection as the differential RU reading between the two flow channels introduce spikes due to a few seconds reading delay (flow channels are in a serial configuration).
Replication	Cryo-EM data collection was done with a single protein batch for samples with and without Ca2+. All other experiments were validated by performing multiple repeats and using different protein purifications.
Randomization	There were no experimental groups requiring randomization.
Blinding	No separate treatment groups were analyzed.

Reporting for specific materials, systems and methods

We require information from authors about some types of materials, experimental systems and methods used in many studies. Here, indicate whether each material, system or method listed is relevant to your study. If you are not sure if a list item applies to your research, read the appropriate section before selecting a response.

Materials & experimental systems

n/a	Involved in the study
☒	☐ Antibodies
☐	☒ Eukaryotic cell lines
☒	☐ Palaeontology and archaeology
☒	☐ Animals and other organisms
☒	☐ Clinical data
☒	☐ Dual use research of concern

Methods

n/a	Involved in the study
☒	☐ ChIP-seq
☒	☐ Flow cytometry
☒	☐ MRI-based neuroimaging

Eukaryotic cell lines

Policy information about cell lines and Sex and Gender in Research

Cell line source(s)	HEK 239 EBNA (Invitrogen)
Authentication	Cells were not authenticated.
Mycoplasma contamination	Cell lines were not tested for mycoplasma contamination.
Commonly misidentified lines (See ICLAC register)	No commonly misidentified lines were used.

March 2021
